# Spatial Differences and Influential Factors of Urban Carbon Emissions in China under the Target of Carbon Neutrality

**DOI:** 10.3390/ijerph19116427

**Published:** 2022-05-25

**Authors:** Kai Liu, Ziyi Ni, Mei Ren, Xiaoqing Zhang

**Affiliations:** 1 College of Geography and Environment, Shandong Normal University, Jinan 250358, China; kailiu@sdnu.edu.cn (K.L.); 201914010232@stu.sdnu.edu.cn (Z.N.); 621071@sdnu.edu.cn (M.R.); 2 Collaborative Innovation Center of Human–Nature and Green Development in Universities of Shandong, Shandong Normal University, Jinan 250358, China

**Keywords:** urban carbon emissions, carbon neutrality, spatial differences, influential factors, exploratory spatial data analysis, Geodetector

## Abstract

Cities are areas featuring a concentrated population and economy and are major sources of carbon emissions (CEs). The spatial differences and influential factors of urban carbon emissions (UCEs) need to be examined to reduce CEs and achieve the target of carbon neutrality. This paper selected 264 cities at the prefecture level in China from 2008 to 2018 as research objects. Their UCEs were calculated by the CE coefficient, and the spatial differences in them were analyzed using exploratory spatial data analysis (ESDA). The influential factors of UCEs were studied with Geodetector. The results are as follows: (1) The UCEs were increasing gradually. Cities with the highest CEs over the study period were located in the urban agglomerations of Beijing–Tianjin–Hebei, Yangtze River Delta, Pearl River Delta, middle reaches of the Yangtze River, and Chengdu–Chongqing. (2) The UCEs exhibited certain global and local spatial autocorrelations. (3) The industrial structure was the dominant factor influencing UCEs.

## 1. Introduction

Humankind is facing a global crisis in the form of climate change that has seriously threatened sustainable economic growth and public health [1,2]. Increased carbon emissions (CEs) owing to human activity have been the main cause of climate change since the 20th century [3,4,5]. China has been the world’s largest carbon emitter since 2006 [6,7]. In the near future, CEs are expected to continue to increase in China [8]. Under this situation, China, as a major and responsible member of the world community, is actively participating in the global governance system. The Chinese government is committed to achieving its carbon peak by 2030 and carbon neutrality by 2060 (http://www.gov.cn/xinwen/2021-09/22/content_5638597.htm, accessed on 6 May 2022).

Cities have a high population density and high intensity of energy consumption, because of which they are a major source of CEs. A total of 85% of direct CEs in China come from cities [9]. The concentration of urban carbon emissions (UCEs) is relatively high in them, with the top 10% of the cities contributing 50% of the total CEs [10,11,12]. The urbanization rate of China exceeded 64% in 2021 and will continue to increase in the future along with CEs. The city is the basic unit used to make policies on reducing CEs and achieving carbon neutrality, and effective response to global climate change requires the comprehensive participation of the city [13,14]. Because of their stage of development, population size, and resource endowments, there are significant differences among cities in China in terms of UCEs [15,16,17,18]. For China, controlling UCEs is critical to achieving its carbon neutrality. Exploring the spatial differences in UCEs and the factors influencing them is crucial to making policies on low-carbon development to achieve carbon neutrality. That is to say, it is necessary to develop differentiated emission reduction policies for cities with different agglomeration types of CEs and targeted policies for the critical factors influencing them.

Previous studies have analyzed the spatial differences in CEs at different regional levels. First, at the international level, Ding and Zhang [19] analyzed the CEs of major countries in the world from multiple perspectives and found that China, the United States, Russia, and Canada were the largest emitters. Qiao et al. [20] forecasted CEs in the countries of the Asia–Pacific Economic Cooperation and concluded that the CEs of 13 countries were rising, whereas those of four countries were falling. Second, at the national level, Li et al. [21] calculated production-based emissions, consumption-based emissions, and emissions transfer at the provincial level in China from 2005 to 2015. Xiong et al. [22] reported that the CEs in China’s regional tourism were growing significantly with marked differences across its regions. Liu et al. [23] found that the intensity of carbon emissions showed a downward trend and was low in the southeast and high in the northwest of China. Yang et al. [24] measured the efficiency of industrial carbon emissions and revealed significant regional differences and varying spatial agglomeration among the provinces of China. Third, in strategic regions, Zhou et al. [25] noted that Macao, Shenzhen, and Hong Kong had the highest intensity of CEs, and cities such as Hong Kong, Shenzhen, Foshan, and Huizhou in the Guangdong–Hong Kong–Macao Greater Bay Area had reached peak emissions. Zhang et al. [26] unraveled the remarkable clustering characteristics of CEs in the Yellow River Economic Belt. Lv et al. [27] posited that Shanghai, Suzhou, and their surrounding cities remain CE hotspots in the Yangtze River Delta urban agglomeration.

Existing studies have also analyzed the effects of relevant factors on CEs. First, the relationship between economic growth and CEs has long been a focus of research. Whether the environmental Kuznets curve (EKC) [28] is applicable to the relationship between them has been an important subject of research. Hundie [29] and Alharthi et al. [30] confirmed the applicability of EKC to their respective issues. Some studies demonstrated M-shaped, N-shaped, U-shaped, and inverted M-shaped patterns in the relationship between economic growth and CEs [31,32,33]. Second, the impact of urbanization on regional CEs has also warranted scholarly attention, and a significant two-way relationship between them has been identified [34,35]. Third, international trade and foreign investment are important channels for economically backward regions to learn advanced production technologies and gain management experience [36]. When foreign investment brings low-carbon technologies and the service industry, it can contribute to the reduction in local emissions [37]. In addition to these factors, land-use efficiency [38], environmental regulation [39], technological progress [40], household income [41], urban morphology [42], and artificial intelligence [43] have also been studied as the influential factors of CEs.

Systematic and in-depth studies have been carried out on the spatial differences and influential factors of CEs at the national and provincial levels. Although some studies have taken cities as the basic unit, most of them have considered specific urban agglomerations and river basins as research objects, which could not be generalized to the entire country. This significantly reduces the reference value of such research for policymaking. If we choose cities as the basic research unit, what are the spatial differences among them in terms of UCEs in China? What are the factors affecting them? To answer these questions, we selected 264 cities at the prefecture level in China as research objects. We used the calculated UCEs from 2008 to 2018 along with exploratory spatial data analysis (ESDA) to explore the spatial differences in UCEs. We also employed Geodetector to examine factors affecting them.

The contributions of this study to the existing research are three-fold: First, most existing studies focused on national and provincial areas, whereas this study takes 264 cities at the prefecture level as the research unit across the country. Second, this study explores the spatial differences in UCEs using ESDA and contributes to revealing the characteristics of spatial correlation. Third, this study also delves into the factors influencing UCEs with Geodetector, which can provide a reference for making policies on urban emission reduction to achieve carbon neutrality.

The remainder of this paper is arranged as follows: Section 2 details the sources of data and the methods used, including the calculation of CEs, ESDA, and Geodetector. Section 3 presents the results of spatial differences and influential factors of UCEs. The conclusions of this study and specific policy implications are provided in Section 4.

## 2. Data Sources and Methods

### 2.1. Data Sources

The consumption of fossil energy is the primary source of CEs in Chinese cities. Drawing on previous studies [44,45], we used the consumption data of 15 energy resources from 264 cities at the prefecture level to calculate the CEs. The data on energy consumption by cities were derived from the statistical yearbook of each city (https://data.cnki.net/, accessed on 17 January 2022), which Chinese government departments provide with authority and credibility. In case data were missing or unavailable, they were collected from the statistical yearbook of the province to which the city belongs (https://data.cnki.net/, accessed on 17 January 2022). The standard energy conversion coefficient (SECC) for coal and the carbon emission coefficient (CEC) for alternative energy resources used in this research referred to the China Energy Statistical Yearbook (https://data.cnki.net/, accessed on 17 January 2022), General Rules for Calculation of the Comprehensive Energy Consumption (GB/T 2589-2020) (http://openstd.samr.gov.cn/, accessed on 17 January 2022), and Guidelines for Compilation of Provincial Greenhouse Gas Inventories (http://www.cbcsd.org.cn/, accessed on 17 January 2022). Table 1 shows the SECC and CEC of different energy resources. The urban economic and social data used to analyze the factors influencing UCEs stemmed from the China City Statistical Yearbook and the China Urban Construction Statistical Yearbook (https://data.cnki.net/, accessed on 17 January 2022).

### 2.2. Methods

#### 2.2.1. Calculation of UCEs

The SECC and CEC were used to calculate the UCEs, as shown in Equation (1):

(1) $$UCEs=\overset{n}{\underset{i=1}{\sum }}\left(K_{i}\times E_{i}\right)$$

UCEs express CEs in a city; $E_{i}$ denotes the consumption of energy *i* after converted to standard coal; $K_{i}$ refers to the CEC of energy *i*. This calculation method for carbon emissions features broad applicability, convenient data acquisition, and consistent statistical caliber [46]. It applies to comparing spatial differences between cities at the prefecture-level in the present study. This method is also widely used in EU countries. In addition to this method, carbon emissions can also be calculated by life cycle assessment and input-output [47], which do not lend themselves to large-scale and continuous comparison.

#### 2.2.2. ESDA

ESDA, proposed by Messner et al. [48], is a method to analyze the spatial structure, spatial form, spatial trend, and outliers contained in data. It is advantageous is exploring the spatial agglomeration and autocorrelation of the given research sample [49,50]. It includes the Global Moran’s *I* and Local Moran’s *I*. The Global Moran’s *I* was used to explore the spatial distribution of UCEs in the entire region. If it is >0, the UCEs have a positive spatial autocorrelation. If it is <0, the research object has a negative spatial autocorrelation. The equations are as follows:

(2) $$I=\frac{\sum_{i=1}^{n}\sum_{j=1}^{n}W_{ij}\left(x_{i}-\bar{x}\right)\left(x_{j}-\bar{x}\right)}{S^{2}\sum_{i=1}^{n}\sum_{j=1}^{n}W_{ij}}$$

(3) $$S=\frac{1}{n}\overset{n}{\underset{i=1}{\sum }}{\left(x_{i}-\bar{x}\right)}^{2}$$

$x_{i}$ and $x_{j}$ represent the CEs of cities *i* and *j*, respectively;$\;\bar{x}$ expresses the average value of UCEs; $W_{ij}$ denotes the spatial weight matrix of cities *i* and *j*. If the two cities have a common boundary, $W_{ij}$ is 1; otherwise, it equals 0. The standardized statistic was used to test the significance as follows:

(4) $$Z\left(I\right)=\frac{\left[1-E\left(I\right)\right]}{\sqrt{Var\left(I\right)}}$$

$Z\left(I\right)$,$\;E\left(I\right)$, and $Var\left(I\right)$ express the significance, mathematical expectation, and variance of Global Moran’s *I*, respectively.

To explore the heterogeneity of UCEs in sub-regions, we resorted to Local Moran’ *I*. The LISA clustering map combined with the Moran scatterplot, and Local Moran’s *I* can reflect the agglomeration types of UCEs. Local Moran’ *I* was defined as:

(5) $$I_{i}=\frac{\sum_{i=1}^{n}\sum_{j=1}^{n}W_{ij}\left(x_{i}-\bar{x}\right)\left(x_{j}-\bar{x}\right)}{S^{2}}$$

Its significance was also tested by Equation (5). By comparing the signs of $Z\left(I\right)$ and the significance of $I_{i}$, the spatial units whose significance levels reach a certain threshold (*p* = 0.05) could be divided into four types, as shown in Table 2. 

#### 2.2.3. Geodetector

Geodetector, proposed by Wang [51], is a statistical analysis method with geographical characteristics and can be used to reveal the factors affecting a given research object [52]. Its biggest advantage lies in that the calculation process and results will not be affected by multivariable collinearity. Suppose the research region consists of sub-regions, and if the variance of the region is greater than its sum in the sub-regions, this accounts for the spatial differentiation between sub-regions. The two variables are spatially connected in case the spatial distribution is consistent. Geodetector can reflect the interaction between different factors and variables and can be used to explore the factors influencing resources and the environment [53,54]. In this paper, Geodetector was used to simulate the factors influencing UCEs. The model was as follows:

(6) $$P_{D,UCEs}=1-\frac{1}{n\sigma_{\mathrm{UCEs}}^{2}}\overset{m}{\underset{i=1}{\sum }}n_{D,i}\sigma_{{\mathrm{UCEs}}_{D,i}}^{2}$$

$P_{D,UCEs}$ expresses the effect of a factor on UCEs. Its range is [0, 1]. If its value is large, this factor has a significant effect. If the value of $P_{D,UCEs}$ is zero, this factor has no relationship with UCEs. A value of 1 indicates that the factor can fully account for the UCEs. *D* refers to the factor affecting UCEs; *n* and $\sigma^{2}$ denote the number and variance of cities; *m* stands for the number of categories of an influential factor; $n_{D,i}$ represents the number of influential factor *D* in class *i*. 

## 3. Results

### 3.1. UCEs

**Figure 1:**
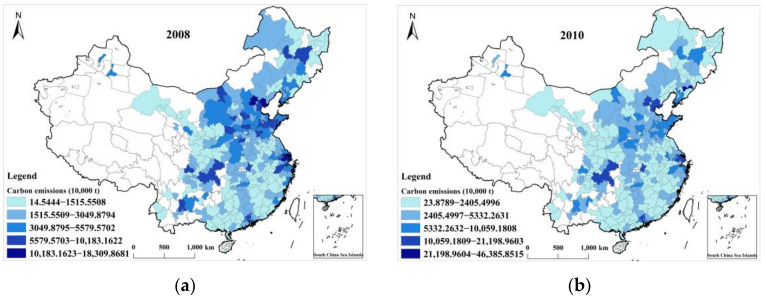

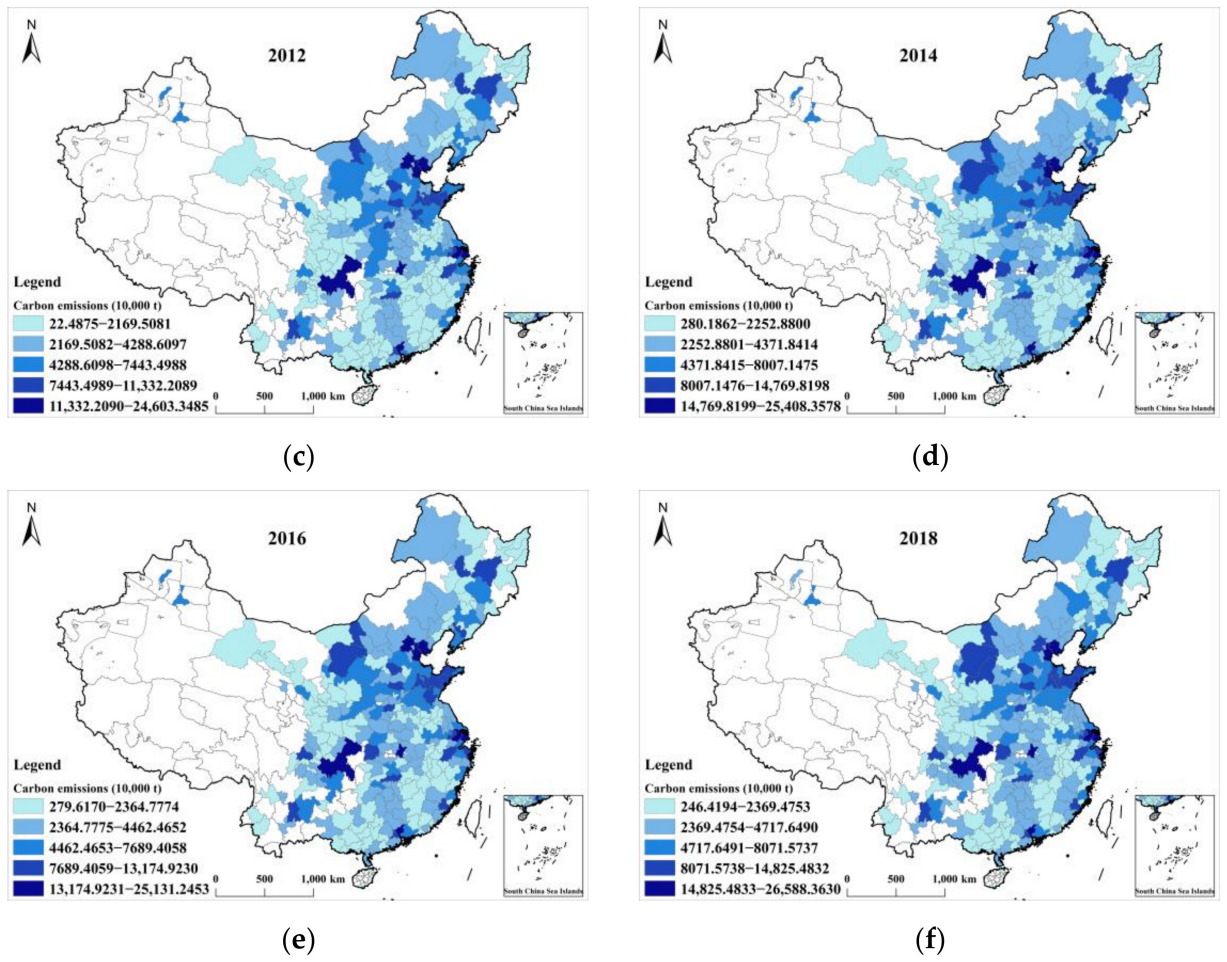
The UCEs in China between 2008 and 2018. (**a**) 2008; (**b**) 2010; (**c**) 2012; (**d**) 2014; (**e**) 2016; (**f**) 2018. Note: This figure shows only the results of even years, and the results of odd years can be seen in Supplementary Materials.

The results showed two significant characteristics of the UCEs in China. First, cities with the highest UCEs over the years were all located in the Beijing–Tianjin–Hebei, Yangtze River Delta, Pearl River Delta, middle reaches of the Yangtze River, and Chengdu–Chongqing urban agglomerations (UA). Figure 1 shows the results of UCEs in China for 2008–2018. The dark colors represent cities with high CEs, whereas light colors represent cities with low CEs. Cities with the largest CEs were all located in the five large UAs mentioned above. These are the most important UAs in China and have strong economic growth and ability, innovation, and a high degree of openness. They are the growth poles that drive China’s economic growth and play a leading role in the national economic system. However, they are also areas with the highest CEs in China and face daunting work for green and low-carbon development.

**Figure 2:**
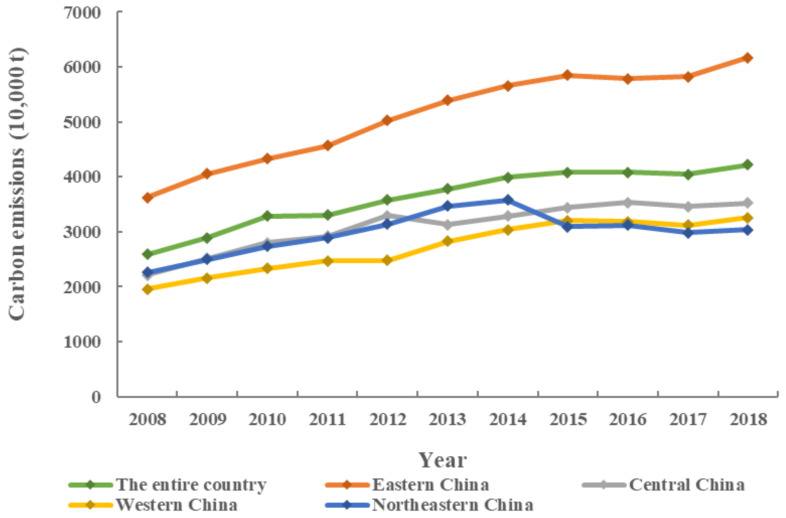
The average values of UCEs in China and its four regions between 2008 and 2018.

Moreover, the UCEs showed a trend of gradually increasing. To make the representation more direct and concise, the average values of UCEs in China and its four regions (Table 3) are used for illustration (Figure 2). From 2008 to 2018, the average value of UCEs in the entire country, and in Eastern, Central, and Western China showed a trend of gradually increasing; only cities in Northeastern China showed a trend of increase from 2008 to 2014 and then decrease in 2015 and 2017. A comparison of the average values of the four regions showed that Eastern China had a much higher value than the other three regions, indicating that it was the key region that requires low-carbon development and emission reduction.

### 3.2. Spatial Differences in UCEs

The global spatial autocorrelation of UCEs in China between 2008 and 2018 was tested using ArcGIS 10.2 software. The results (Table 4) demonstrated that the Global Moran’s *I* was positive every year and passed the 1% significance test. This illustrates that the UCEs in China had similar spatial characteristics of agglomeration. However, the Global Moran’s *I* was not large—its highest value was only 0.2602—which indicates that the global spatial autocorrelation of UCEs in China was not very significant.

**Figure 3:**
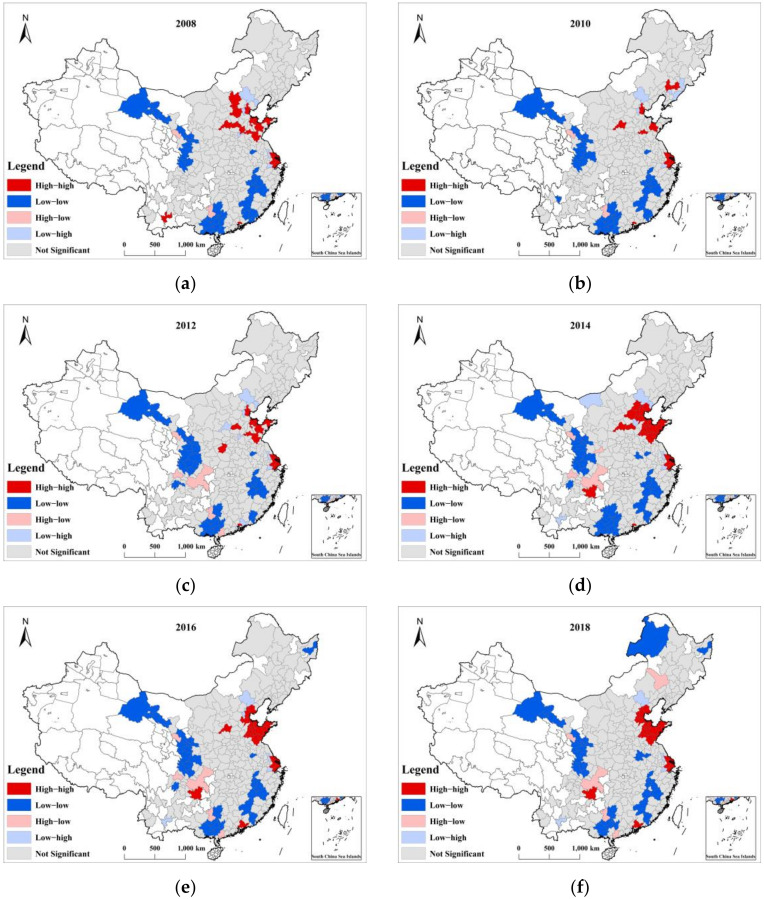
The local spatial autocorrelation of UCEs in China between 2008 and 2018. (**a**) 2008; (**b**) 2010; (**c**) 2012; (**d**) 2014; (**e**) 2016; (**f**) 2018. Note: This figure shows only the results of even years, and the results of odd years can be seen in Supplementary Materials.

The local spatial autocorrelation of UCEs in China between 2008 and 2018 was also tested by ArcGIS 10.2 software. The UCEs showed some local spatial autocorrelation, which could be divided into four types (Figure 3): High-high, high-low, low-low, and low-high. However, compared with the number of cities with insignificant local spatial autocorrelation, the proportion of these four types of cities was not high. In 2017, the number of these four types of cities was the highest, accounting for only 27% of the total. They were the fewest, accounting for only 20.5% in 2010. The number of cities in each type was stable. The low-low type had the largest number of cities, and the high-low type had the least. Low-low-type cities were mainly distributed in Guangdong, Fujian, Gansu, and Jiangxi provinces. High-high-type cities were mainly distributed in Shandong Province, Hebei Province, around Shanghai, and Guangdong Province. Both low-low-type and high-high-type cities were identified in Guangdong Province, which showed prominent differences in UCEs.

Previous studies on spatial differences of CEs in China concluded that the carbon emissions in China showed spatial autocorrelation [21,22]. The hot spot area was located in the North China Plain, and the cold spot area was located in the northwest [23]. However, our research is specific to the city level, which can unravel which city belongs to the hot spot area and which city belongs to the cold spot area, thereby providing more accurate information for policymaking in emission reduction.

### 3.3. Influential Factors of UCEs

With reference to the previous studies [55,56] and the characteristics of urban development in China, nine indicators—economic scale, industrial structure, population urbanization, land urbanization, resident income, technological progress, energy structure, openness, and environmental regulation—were considered as the factors influencing UCEs. The reasons are as follows:

(1) Economic scale. It is expressed by urban GDP. The expansion of economic scale increases the output of products, whereas the expansion of output may increase CEs at the same technological level. When economic growth reaches a certain stage, that is, after the turning point of the EKC, the expansion of the economic scale may lead to a reduction in CE changes in the technology level and production model [57].

(2) Industrial structure. It is expressed by the proportion of the added value of the secondary industry. The industrial structure upgrading is an important indicator of economic growth. Different industrial structures have different effects on CEs [58]. A stage dominated by the secondary industry is inevitable in the evolution of the industrial structure. The industrial and transportation sectors in the secondary industry are important sources of CEs. With the development of the industry, the expansion in the production scale brings about more CEs.

(3) and (4) Population urbanization and land urbanization. They are expressed by the proportions of urban population and land for construction, respectively. The urban population is growing, and the land for urban construction is expanding. The demand for energy will continue to increase with rising urbanization and may lead to a corresponding increase in CEs. When the urbanization rate exceeds a certain level, the application of new environmental protection technology and improvements in energy efficiency will be conducive to reducing CEs [59].

(5) Resident income. It is expressed by the average wage of urban workers. The awareness of energy conservation and emission reduction among residents and enterprises improves with the increase in the urban resident income, which is conducive to reducing CEs [60]. However, after the increase in urban resident income, local demand increases to scale up the manufacture of products, which increases CEs.

(6) Technological progress. It is expressed by the expenditure on science and technology. On the one hand, environmentally friendly technological progress can improve energy efficiency in the production process. On the other hand, technological progress may not be environmentally friendly, and the expansion of the production scale accompanied by technological progress may increase CEs [61].

(7) Energy structure. It is expressed by the proportion of coal consumption. It can directly determine the intensity of CEs. When the proportion of clean and new energy is high, CEs at the same level of energy consumption are lower. While the chemical energy dominated by coal is the main source of CEs, the high proportion of coal is not conducive to reducing CEs [62].

(8) Openness. It is expressed by the amount of foreign capital used. Opening up is conducive to attracting foreign enterprises that can help save energy and reduce emissions to carry out more green production, thus reducing CEs [63]. At the same time, foreign capital can flow in owing to low environmental standards and intensity of environmental regulation in developing countries, which can lead to the pollution haven effect and problem of growing CEs. 

(9) Environmental regulation. It is expressed by the investment in environmental pollution control. A higher intensity of regulation forces enterprises to reduce emissions [64,65]. Therefore, the intensity of environmental regulation is an important factor restraining CEs by enterprises.

**Figure 4:**
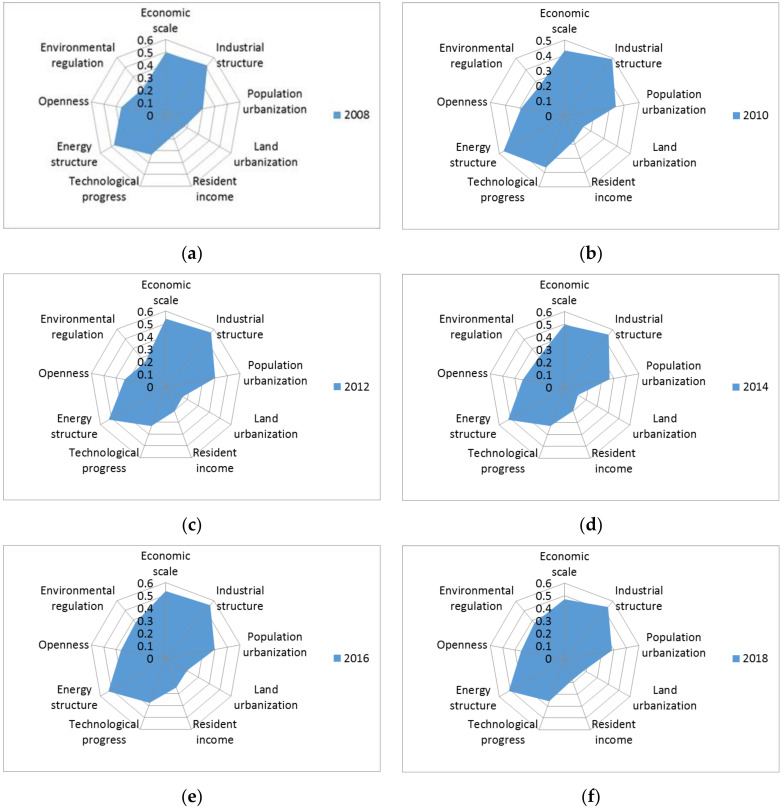
The influential factors of UCEs in China from 2008 to 2018. (**a**) 2008; (**b**) 2010; (**c**) 2012; (**d**) 2014; (**e**) 2016; (**f**) 2018. Note: All the results passed the 1% significance test. This figure shows only the results of even years, and the results of odd years can be seen in Supplementary Materials.

Figure 4 shows the Geodetector results for UCEs in China between 2008 and 2018. The contribution of the industrial structure to UCEs was higher than other factors every year. The industrial structure dominated by the secondary industry was the dominant factor affecting UCEs in China. The economic scale and energy structure also had a relatively significant effect on UCEs. The impact of land urbanization, resident income, and environmental regulation on UCEs was not significant. From the evolution of the industrial structure in China, industrialization has entered a stage of rapid development; the total industrial output has continued to rise; a complete industrial system has been established; China has become a manufacturer for the world. China has now entered the late stage of industrialization, which brings about the problems of high energy consumption and CEs. In 2017, for example, the energy consumption per unit GDP in China was 152 g of standard oil per US dollar, 25 g/US dollars higher than the world’s average and more than twice that of the UK. Therefore, of all influential factors, the industrial structure was the leading driver of UCEs in China.

Some studies have found that the population size or economic scale is the main factor affecting carbon emissions [16,22]. This is not consistent with our findings that different research objects may cause this difference.

## 4. Conclusions and Policy Implications

### 4.1. Conclusions

This study attempted to explore the spatial differences and influential factors of UCEs in China. In total, 264 cities at the prefecture level in China from 2008 to 2018 were taken as research objects. ESDA was used to analyze the spatial differences in UCEs, and Geodetector was employed to analyze the factors affecting UCEs. The conclusions were as follows: (1) The UCEs showed a gradual upward trend, and cities with the highest emissions were located in the Beijing–Tianjin–Hebei, Yangtze River Delta, Pearl River Delta, middle reaches of the Yangtze River, and Chengdu–Chongqing UAs. (2) The UCEs had significant spatial differences with certain global and local spatial autocorrelations. (3) The industrial structure had become the most dominant factor affecting UCEs. 

We used ESDA alone to analyze the spatial differences of UCEs. In the future, it is necessary to further study the spatial differences of UCEs using Dagum’s decomposition of the Gini coefficient, kernel density estimation, and standard deviational ellipse. Our analysis of the influential factors of CEs focused on the macro socio-economic aspect. How micro factors such as individual heat sources and communication affect CEs merits more attention in the future.

### 4.2. Policy Implications

(1) Due to spatial differences in the UCEs in China, cities with the highest CEs are from the five national UAs. UAs should thus be the key areas for China to make policies on emissions reduction. China has five national UAs, nine medium UAs, and six small UAs. These twenty UAs are regions with the most concentrated economies and populations and the highest level of industrialization and urbanization in China. They have thus become regions with the most concentrated CEs. The UAs have established a relatively sound infrastructure and an integrated mechanism of market and basic public services. They also need a cooperative mechanism of low-carbon development. This can improve their threshold of industrial access, restrict industries with high energy consumption and emissions, and help build an integrated mechanism of low-carbon governance. The UAs are also the most developed areas in science and technology. These advantages need to be used to develop technologies that can save energy and provide technical support for reducing CEs.

(2) Due to the spatial autocorrelation of the UCEs in China, an “urban carbon reduction community” should be established. First, a community of all high-high-type cities should be established. These are cities with large and concentrated CEs that need to control the expansion of their industries that are high emitters, formulate emissions standards for these industries, and require them to implement the relevant standards. In addition, such cities need to optimize their industrial spatial layout and encourage their high-carbon industries to reduce emissions through orderly regional transfer, clustering development, transformation, and upgrades while conforming to national industrial policies. For low-low-type cities that emit carbon, it is necessary to establish a low-carbon mode of industry, construction, transportation, and energy system and advocate a green and low-carbon lifestyle and mode of consumption. Low-carbon industrial demonstration parks can be built in such cities to support the construction of a low-carbon city.

(3) As the industrial structure has become the dominant factor affecting UCEs, its continual optimization should be promoted. All cities need to use the development of ecological civilization and green development as the basic guidance and promote low-carbon technology and modes of clean production in various industries and sectors. Energy conservation and environmental protection have the advantages of a long industrial chain, driving employment, energy conservation, and emission reduction. Cities with such prerequisites need to develop energy conservation and environmental protection. For cities dominated by the secondary industry, advanced technologies need to be used to improve the traditional manufacturing industry, formulate emissions reduction targets for high-emission industries, and control CEs. For energy conservation, a system for the control and evaluation of energy consumption should be established to promote energy conservation in power, steel, building materials, nonferrous materials, and chemical industries while promoting energy saving in new buildings and transportation. For cities in which the tertiary industry is the leading industry, it is necessary to develop strategic emerging industries and service industries and use representative commercial institutions such as shops and hotels as pilot projects to implement supply chain management to reduce emissions.

## Figures and Tables

**Table 1:** The SECC and CEC of different energy resources.

Energy	SECC	CEC	Energy	SECC	CEC
Raw coal	0. 7143	2. 492	Fuel oil	1. 4286	2. 219
Cleaned coal	0. 9000	2. 631	Liquified petroleum gas	1. 7143	1. 828
Coal products	0. 6000	2. 631	Natural gas	1. 2143	2. 162
Coke	0. 9714	2. 977	Liquified natural gas	1. 7572	2. 660
Crude oil	1. 4286	2. 104	Refinery gas	1. 5714	1. 654
Gasoline	1. 4714	1. 988	Coke oven gas	0. 6143 kgce /m^3^	1. 288
Kerosene	1. 4714	2. 051	Blast furnace gas	0. 1286 kgce /m^3^	7. 523
Diesel oil	1. 4571	2. 167			

**Table 2:** Four types of local spatial autocorrelation.

Type	$Z\left(I\right)$	$I_{i}$	Explanation
High−high	>0	Significantly positive	The CEs of this city and its adjacent cities are relatively high; that is, it is a hotspot.
Low−low	<0	Significantly positive	The CEs of this city and its adjacent cities are relatively low; that is, it is a cold spot.
High−low	>0	Significantly negative	Cities with high CEs are surrounded by cities with low emissions.
Low−high	<0	Significantly negative	Cities with low CEs are surrounded by those with high emissions.

**Table 3:** The four regions in China.

Regions	Provinces (Municipality Directly under the Central Government, Autonomous Region)
Eastern China	Beijing, Tianjin, Hebei, Shanghai, Jiangsu, Zhejiang, Fujian, Shandong, Guangdong, and Hainan.
Central China	Shanxi, Anhui, Jiangxi, Henan, Hubei, and Hunan.
Western China	Inner Mongolia, Guangxi, Chongqing, Sichuan, Guizhou, Yunnan, Tibet, Shaanxi, Gansu, Qinghai, Ningxia, and Xinjiang.
Northeastern China	Liaoning, Jilin, and Heilongjiang.

**Table 4:** The global spatial autocorrelation of UCEs in China between 2008 and 2018.

Year	Moran’s *I*	*Z*	*p*-Value
2008	0.2400	5.8676	0.001
2009	0.2400	5.8426	0.001
2010	0.2165	5.2357	0.001
2011	0.2321	5.6237	0.001
2012	0.2355	5.6809	0.001
2013	0.2540	6.1822	0.001
2014	0.2562	6.2269	0.001
2015	0.2602	6.2882	0.001
2016	0.2483	5.9471	0.001
2017	0.2497	5.9488	0.001
2018	0.2502	5.9493	0.001

## Data Availability

The data presented in this study are available on request from the corresponding author. The data are not publicly available because research is ongoing.

## Supplementary Materials

The following supporting information can be downloaded at: https://www.mdpi.com/article/10.3390/ijerph19116427/s1, Supplementary Materials Figure S1: The results of calculation of UCEs in China between 2008 and 2018, Figure S2: Results of local spatial autocorrelation of UCEs in China between 2008 and 25518 and Figure S3: Results of the influential factors of UCEs in China from 2008 to 2018. Note: All the results passed the 1% significance test.
