# Supplementary material for: Spatial Differences and Influential Factors of Urban Carbon Emissions in China under the Target of Carbon Neutrality
Source: Int J Environ Res Public Health. 2022 May 25;19(11):6427. doi: 10.3390/ijerph19116427 (PMC9180286; doi:10.3390/ijerph19116427)
Supplement: Supplementary file 1 [file ijerph-19-06427-s001.zip › ijerph-1702663-supplementary.pdf]

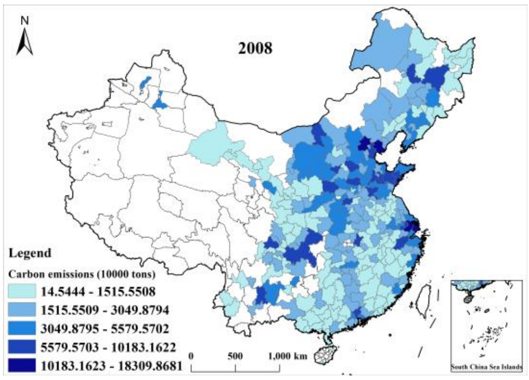
N
2008
Legend
Carbon emissions (10000 tons)
14.5444 - 1515.5508
1515.5509 - 3049.8794
3049.8795 - 5579.5702
5579.5703 - 10183.1622
10183.1623 - 18309.8681
0 500 1,000 km
South China Sea Islands

(a)

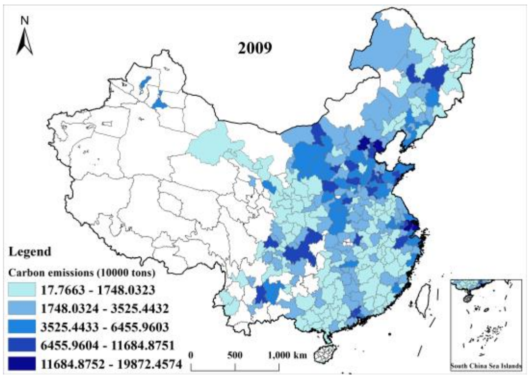
N
2009
Legend
Carbon emissions (10000 tons)
17.7663 - 1748.0323
1748.0324 - 3525.4432
3525.4433 - 6455.9603
6455.9604 - 11684.8751
11684.8752 - 19872.4574
0 500 1,000 km
South China Sea Islands

(b)

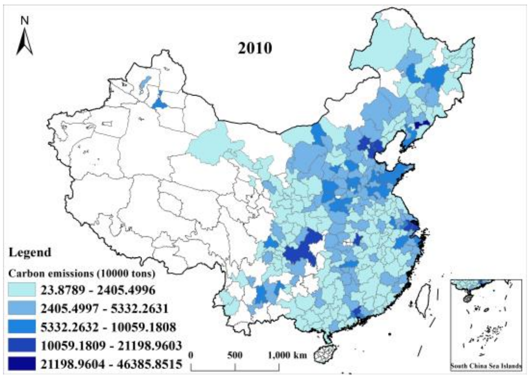
N
2010
Legend
Carbon emissions (10000 tons)
23.8789 - 2405.4996
2405.4997 - 5332.2631
5332.2632 - 10059.1808
10059.1809 - 21198.9603
21198.9604 - 46385.8515
0 500 1,000 km
South China Sea Islands

(c)

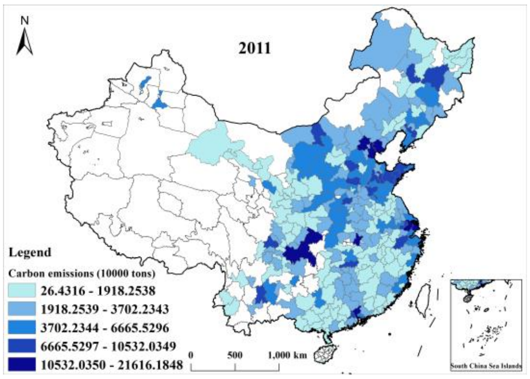
N
2011
Legend
Carbon emissions (10000 tons)
26.4316 - 1918.2538
1918.2539 - 3702.2343
3702.2344 - 6665.5296
6665.5297 - 10532.0349
10532.0350 - 21616.1848
0 500 1,000 km
South China Sea Islands

(d)

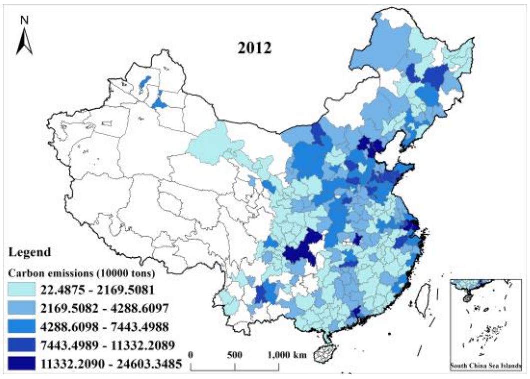
N
2012
Legend
Carbon emissions (10000 tons)
22.4875 - 2169.5081
2169.5082 - 4288.6097
4288.6098 - 7443.4988
7443.4989 - 11332.2089
11332.2090 - 24603.3485
0 500 1,000 km
South China Sea Islands

(e)

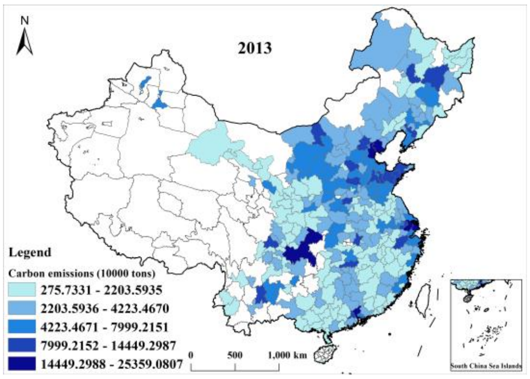
N
2013
Legend
Carbon emissions (10000 tons)
275.7331 - 2203.5935
2203.5936 - 4223.4670
4223.4671 - 7999.2151
7999.2152 - 14449.2987
14449.2988 - 25359.0807
0 500 1,000 km
South China Sea Islands

(f)

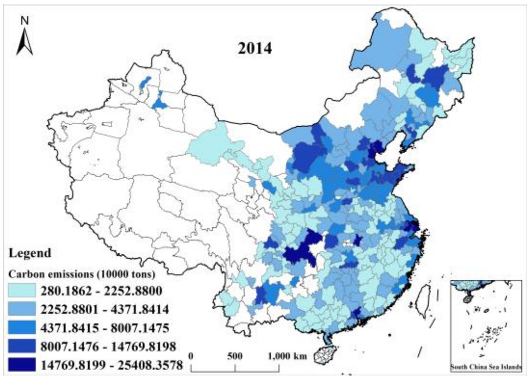
N
2014
Legend
Carbon emissions (10000 tons)
280.1862 - 2252.8800
2252.8801 - 4371.8414
4371.8415 - 8007.1475
8007.1476 - 14769.8198
14769.8199 - 25408.3578
0 500 1,000 km
South China Sea Islands

(g)

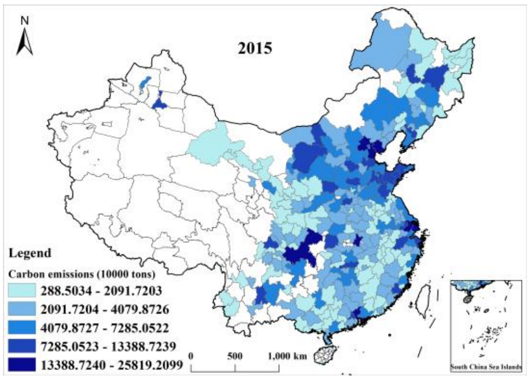
N
2015
Legend
Carbon emissions (10000 tons)
288.5034 - 2091.7203
2091.7204 - 4079.8726
4079.8727 - 7285.0522
7285.0523 - 13388.7239
13388.7240 - 25819.2099
0 500 1,000 km
South China Sea Islands

(h)

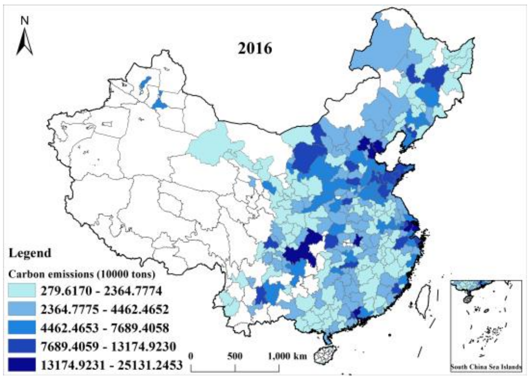

(i)

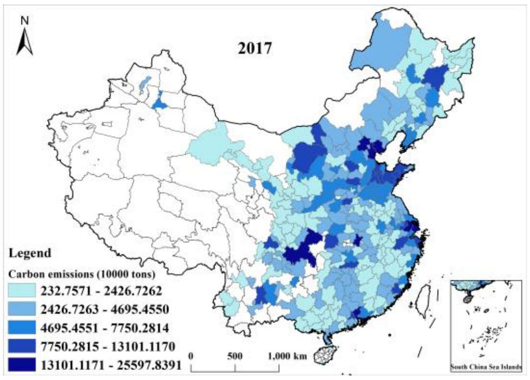

(j)

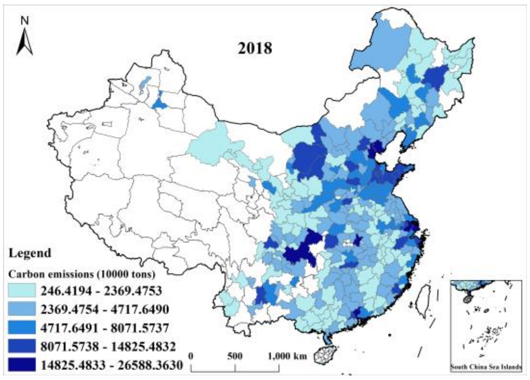

(k)

Figure S1. The results of calculation of UCEs in China between 2008 and 2018.

(a) UCEs in 2008; (b) UCEs in 2009; (c) UCEs in 2010; (d) UCEs in 2011; (e) UCEs in 2012; (f) UCEs in 2013; (g) UCEs in 2014; (h) UCEs in 2015; (i) UCEs in 2016; (j) UCEs in 2017; (k) UCEs in 2018.

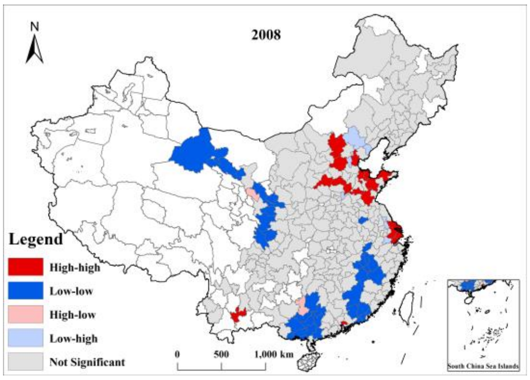

(a)

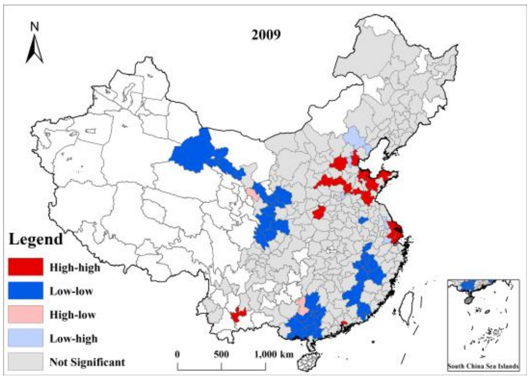

(b)

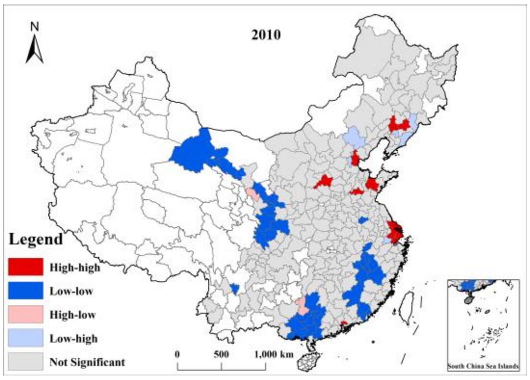
N
2010
Legend
High-high
Low-low
High-low
Low-high
Not Significant
0 500 1,000 km
South China Sea Islands

(c)

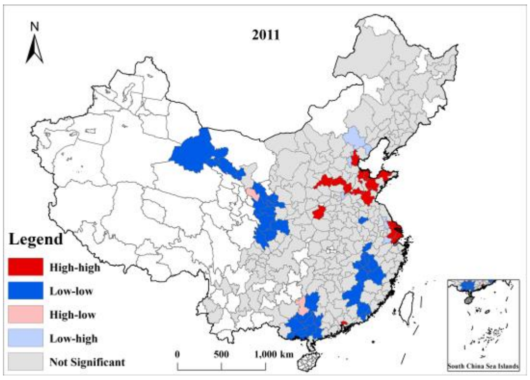
N
2011
Legend
High-high
Low-low
High-low
Low-high
Not Significant
0 500 1,000 km
South China Sea Islands

(d)

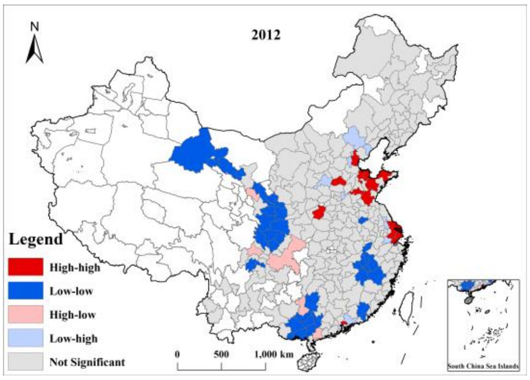
N
2012
Legend
High-high
Low-low
High-low
Low-high
Not Significant
0 500 1,000 km
South China Sea Islands

(e)

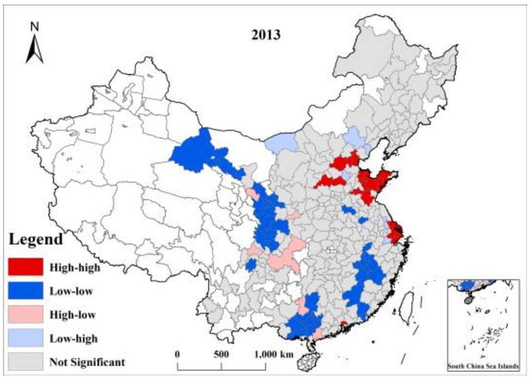
N
2013
Legend
High-high
Low-low
High-low
Low-high
Not Significant
0 500 1,000 km
South China Sea Islands

(f)

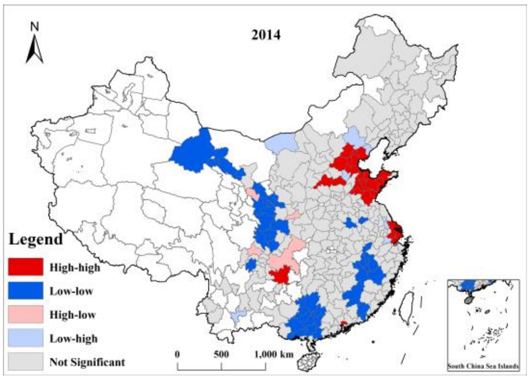
N
2014
Legend
High-high
Low-low
High-low
Low-high
Not Significant
0 500 1,000 km
South China Sea Islands

(g)

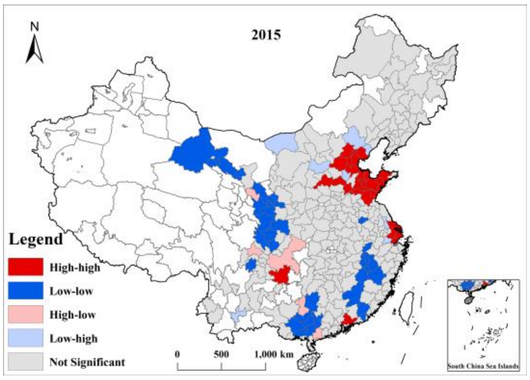
N
2015
Legend
High-high
Low-low
High-low
Low-high
Not Significant
0 500 1,000 km
South China Sea Islands

(h)

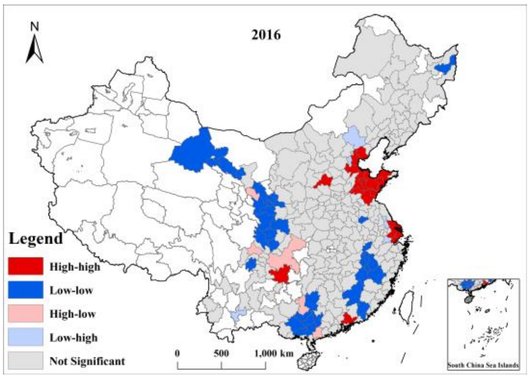
N
2016
Legend
High-high
Low-low
High-low
Low-high
Not Significant
0 500 1,000 km
South China Sea Islands

(i)

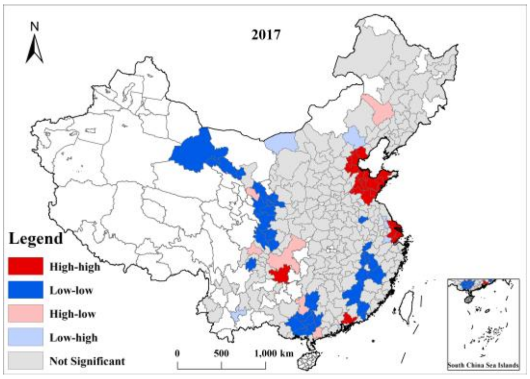
N
2017
Legend
High-high
Low-low
High-low
Low-high
Not Significant
0 500 1,000 km
South China Sea Islands

(j)

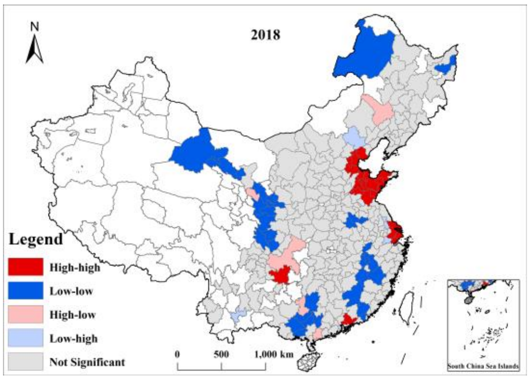

(k)

Figure S2. Results of local spatial autocorrelation of UCEs in China between 2008 and 2018.

(a) Local spatial autocorrelation in 2008; (b) Local spatial autocorrelation in 2009; (c) Local spatial autocorrelation in 2010; (d) Local spatial autocorrelation in 2011; (e) Local spatial autocorrelation in 2012; (f) Local spatial autocorrelation in 2013; (g) Local spatial autocorrelation in 2014; (h) Local spatial autocorrelation in 2015; (i) Local spatial autocorrelation in 2016; (j) Local spatial autocorrelation in 2017; (k) Local spatial autocorrelation in 2018.

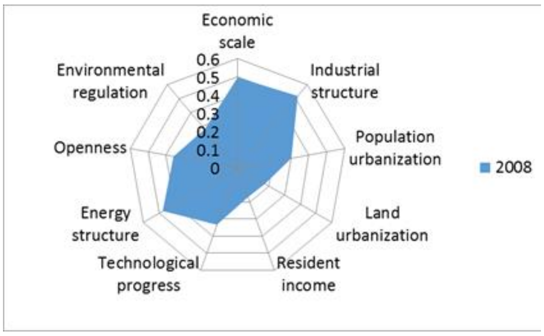

(a)

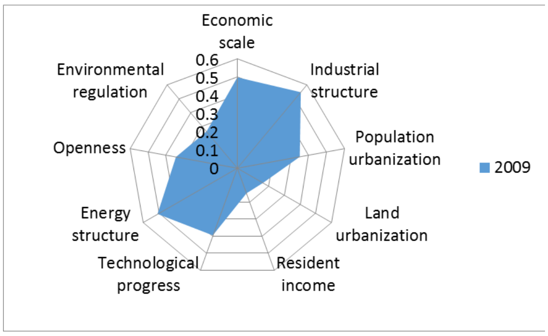

(b)

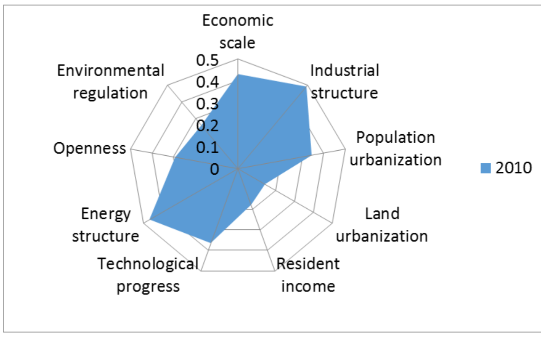

(c)

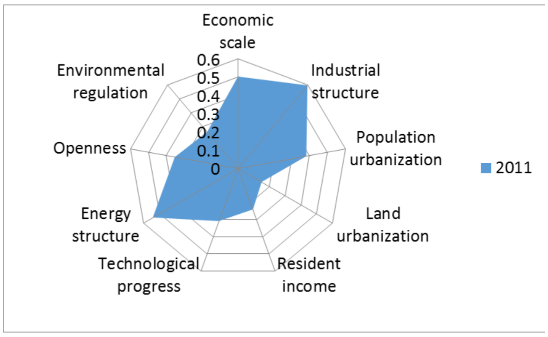

(d)

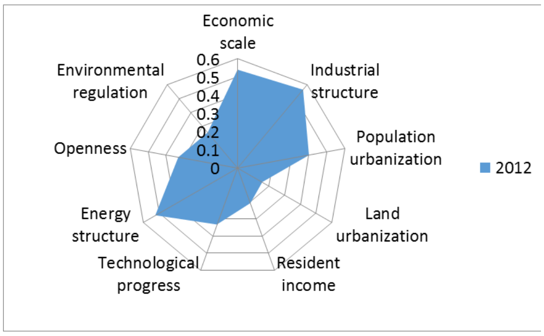

(e)

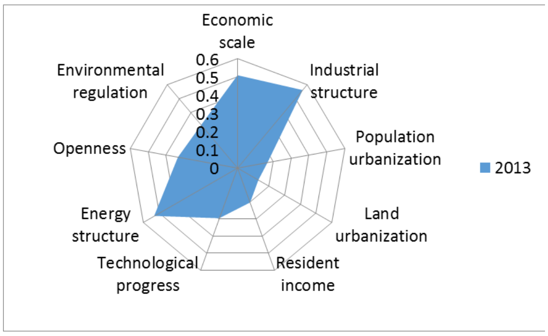

(f)

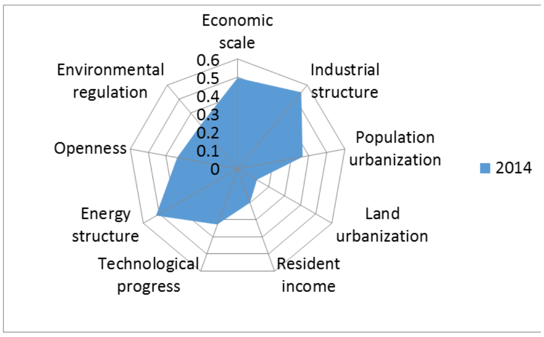

(g)

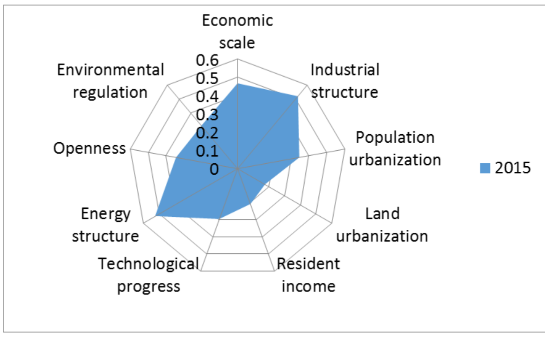

(h)

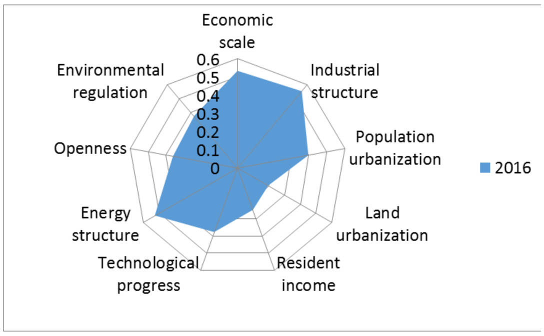

(i)

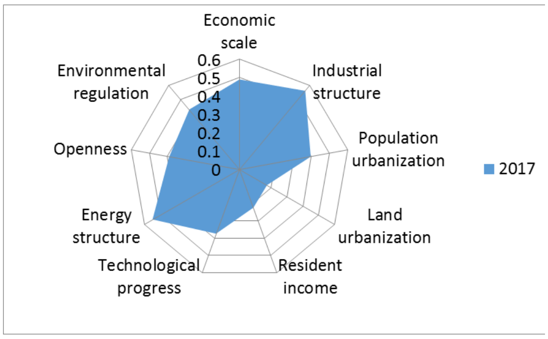

(j)

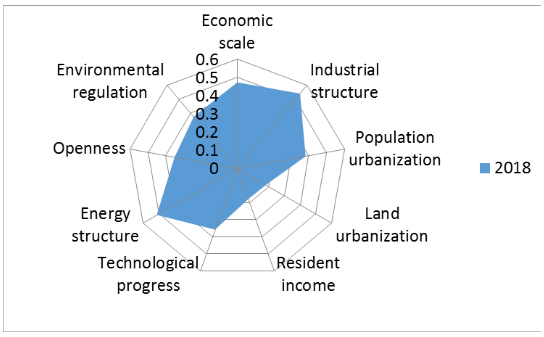

(k)

Figure S3. Results of the influential factors of UCEs in China from 2008 to 2018. Note: All the results passed the 1% significance test.

(a) Influential factors in 2008; (b) Influential factors in 2009; (c) Influential factors in 2010; (d) Influential factors in 2011; (e) Influential factors in 2012; (f) Influential factors in 2013; (g) Influential factors in 2014; (h) Influential factors in 2015; (i) Influential factors in 2016; (j) Influential factors in 2017; (k) Influential factors in 2018.
